# Radiotherapy enhances responses of lung cancer to CTLA-4 blockade

**DOI:** 10.1186/s40425-019-0542-z

**Published:** 2019-03-06

**Authors:** Anna Wilkins, Fiona McDonald, Kevin Harrington, Alan Melcher

**Affiliations:** 1 The Royal Marsden NHS Foundation Trust, Fulham Road, London, SW3 6JJ UK; 2 Division of Radiotherapy and Imaging, The Institute of Cancer Research, 123 Old Brompton Road, London, SW7 3RP UK

**Keywords:** Abscopal effect, CTLA-4 blockade, Ipilimumab, Non-small cell lung cancer

## Abstract

Formenti et al. have recently reported the clinical outcomes and translational readouts of a trial of the anti-CTLA-4 inhibitor, ipilimumab, in combination with palliative radiotherapy in 39 patients with non-small cell lung cancer. A radiological response was seen in 18% of patients and 31% of patients experienced disease control. These clinical outcomes appear to be superior to historical studies using ipilimumab alone and suggest that radiation may have triggered systemic, so-called abscopal, immune responses in some patients. Induction of interferon-beta (IFN-β) and maximal expansion and contraction of distinct T cell receptor clones were the most significant factors predicting response. Importantly, established predictive biomarkers of response to immunotherapy alone, including the expression of PD-L1 in diagnostic biopsies and tumour mutational burden, did not predict response. The report provides important human qualification of pre-clinical mechanistic insights indicating that abscopal responses can be generated with optimised radiotherapy fractionation schedules and anti-CTLA-4 inhibition. Additionally, an intriguing mechanism by which radiation can be immunogenic is described, namely radiation-induced transcriptional upregulation of neo-antigens.

## Main text

Tumour shrinkage at a distance from radiotherapy portals, in the form of the abscopal response, has, historically, been rarely observed. The discovery of immune checkpoint inhibitors (ICI) has raised the possibility that abscopal responses could be induced more commonly by combining radiation with ICI. Formenti and colleagues have recently reported clinical outcomes and translational readouts from a trial that seeks to address this question using anti-CTLA-4 immunotherapy and palliative radiation in patients with non-small cell lung cancer (NSCLC) [1]. Thirty-nine patients with metastatic NSCLC received four cycles of ipilimumab with radiotherapy administered between days one and five of the first ipilimumab treatment. Recruited patients had progressed through at least one previous systemic treatment and had a substantial burden of metastatic disease; 41% of patients had pre-existing brain metastases. Over a decade of pre-clinical experimentation by the authors provided a sound scientific basis for the chosen fractionation schedules of 3 × 9.5 Gy delivered over three days and 5 × 6 Gy delivered over five days. Both schedules closely mirror those showing maximal synergy with anti-CTLA-4 immunotherapy in murine models [2] although no human studies have been undertaken to establish these schedules as optimal in combination with immunotherapy in the clinic.

Twenty-one of 39 patients completed all four cycles of ipilimumab and could be evaluated for response by RECIST criteria on day 88 following commencement of ipilimumab. Unfortunately, eighteen patients could not be evaluated at day 88, predominantly due to either disease progression or death beforehand. Of the evaluable patients, 7/21 (33%) showed a radiological response and a further five patients showed stable disease – representing a disease control rate of 12/39 (31%) of all patients. The median overall survival in patients with disease control was 20.4 months (95% CI: 12.9 months to not reached) compared with 3.5 months (95% CI: 3.1–7.4 months) in patients who did not achieve disease control (log-rank test *P* < 0.001). The authors noted that radiotherapy did not add additional toxicity beyond that associated with ipilimumab alone.

Rigorous evaluation of the abscopal effect when using immunotherapy/radiotherapy combinations requires randomisation of patients to immunotherapy alone versus radiotherapy and immunotherapy given together. Therefore, in the current non-randomised study, it is impossible to know how much of the observed benefit was due to ipilimumab alone and how much was contributed by a radiation-induced abscopal effect. Nonetheless, the favourable clinical responses seen in the current study can be compared with the disappointing historical studies of CTLA-4 inhibitors, both as single-agents and combined with chemotherapy, in metastatic non-small cell lung cancer [3, 4]. A recent systematic review and meta-analysis of immunotherapy in NSCLC found no statistically significant improvement in overall survival for anti-CTLA-4 inhibitors [5]. In the light of these poor responses, the rate of disease control of 31% seen by Formenti et al. suggests that a radiation-induced abscopal effect may be occurring in some patients.

An impressive range of biological profiling using both tumour tissue and blood samples was conducted alongside the clinical trial, and these findings are particularly interesting. Neither PD-L1 expression, an established predictive marker of response to immunotherapy in NSCLC [6], nor CD8+ T cell infiltration in pre-treatment diagnostic biopsies showed any association with treatment response. Instead, analysis of circulating soluble markers and immune cells at baseline indicated that the absolute lymphocyte count was lower and the regulatory T cell count was higher in responding patients versus those with stable or progressive disease.

Longitudinal analysis involved evaluation of changes in immune cells and serum markers occurring between baseline and day 22 of treatment, i.e. shortly after radiotherapy completion. Two known pharmacodynamic markers for anti-CTLA-4 response, namely ICOS expression on CD4 T cells and proliferation of CD8 and CD4 T cells, increased across the majority of patients irrespective of response. Other soluble circulating markers evaluated included major histocompatibility complex class I chain-related proteins A and B (sMICA/sMICB), neither of which showed any association with therapeutic benefit at baseline or longitudinally.

In a pattern that closely mirrors findings in pre-clinical murine models, there was a strong association between change in serum IFN-β (between baseline and day 22 of treatment) and clinical response. The seven patients with a radiological response showed the largest rise in IFN-β. In contrast, the 23 evaluable patients with progressive disease (including those who did not complete ipilimumab treatment) showed no significant rise in IFN-β at day 22 of treatment. An intermediate rise in IFN-β occurred in patients with stable disease. Random forest classification identified change in IFN-β as the most significant predictor of response of all biological parameters analysed. According to the pre-clinical models, such IFN-β is produced following radiation-induced entry of double-stranded DNA into the cytosol and subsequent activation of the cGAS/STING pathway. IFN-β may be produced as both a tumour-cell intrinsic response or from antigen presenting cells such as BATF3-dependent dendritic cells [7, 8].

Deep sequencing of the T cell receptor (TCR) CDR3 region (TCR Seq) in peripheral blood samples led to identification of a second biological parameter that significantly predicted clinical response. Intriguingly, a specific TCR dynamic was seen whereby responders showed a significantly larger increase in both the expansion and contraction of different TCR clones than non-responders. To investigate the tumour specificity of these TCR clonal dynamics seen in the blood, the authors then explored the TCR Seq profile of tumour-infiltrating lymphocytes (TIL-TCR) from four patients who showed a varied response to radiation plus ipilimumab. Sequencing of TIL-TCR showed that the number of tumour-specific clones expanding and persisting in the blood was substantially higher in the patient showing complete response than in the three other patients with either a less marked clinical response or disease progression.

The selection of four patients with varied clinical response for comprehensive tumour profiling is likely to relate to the size of residual tumour samples. Diagnostic biopsies of lung tumours are often very small meaning that molecular profiling can be challenging. Despite this, the authors were able to perform whole exome sequencing (WES) alongside the TIL-TCR sequencing described earlier. WES demonstrated a varied mutational load across the four samples. However, neither mutational load nor predicted number of neo-antigens showed any correlation with clinical response. Additionally, the predicted MHC-I binding affinity did not differ significantly between predicted neo-epitopes. Finally, no specific mutations were identified in the frequently mutated *TAP or B2M* genes or in genes in the interferon pathway. In summary, WES did not identify predictors of clinical response, which, once again, is in contrast to established findings using ICI without radiotherapy [9].

Next, the authors further explored the relationship between neo-epitopes and TIL-TCR Seq and uncovered a fascinating novel mechanism of radiation-induced immunogenicity. Two neo-epitopes that occurred in the patient with complete response were both derived from the same single mutation but bound to different HLA loci. The mutation lies in the KPNA2 gene, which the authors demonstrated was upregulated by radiotherapy in a patient-derived lung cancer xenograft. TCR clones reacting to this mutation were almost entirely absent before radiation but showed a dramatic expansion in peripheral blood samples after radiation.

Radiation-induced cell lysis can release existing intracellular neo-antigens and radiation can induce new mutations via direct damage to DNA. This work demonstrates a third distinct mechanism by which radiation can be immunogenic, namely radiation-induced upregulation of pre-existing neo-antigens. To our knowledge, this upregulation of neo-antigens by radiotherapy that triggers new tumour-specific TCR clones has not been demonstrated previously. Recent pre-clinical studies have shown that radiotherapy causes a broadening of the TCR repertoire [10], which may be important for the observed synergy with ICI. As radiotherapy is known to cause extensive transcriptional upregulation, it is possible that unmasking of pre-existing neo-antigens occurs with this upregulation, which contributes to the broadening of the TCR repertoire described above. The precise impact of radiotherapy on the induction and upregulation of neo-antigens is an area in need of further study – including longitudinal genomic and proteomic profiling in a human context.

There are many unanswered questions about the abscopal effect, which remains a somewhat elusive phenomenon. For example, is irradiation of the primary tumour, as opposed to metastatic sites, necessary? Does radiation need to be directed to lesions above a threshold size? Additionally, should the regional draining lymph nodes be included in or excluded from the radiation field? A number of randomised clinical trials currently seek to address these questions. In the meantime, this fascinating report indicates the potential for meaningful abscopal responses with ipilimumab and radiotherapy despite the considerable intra-tumoural heterogeneity of metastatic lung cancer. The study also reinforces the importance of embedding high quality translational science within clinical trials. Here, Formenti and colleagues provide elegant validation of pre-clinical insights about the importance of type 1 interferon induction in a human context. Finally, there is widespread transcriptional upregulation in response to radiotherapy and it will be exciting to explore further how radiation can upregulate neo-antigens in future studies.

## Abbreviations

ICI: Immune checkpoint inhibition
IFN-β: Interferon-beta
MICA: Major histocompatibility class I chain-related protein A
MICB: Major histocompatibility class I chain-related protein B
NSCLC: Non-small cell lung cancer
TCR: T cell receptor
TCR-Seq: T cell receptor sequencing
TIL-TCR: T cell receptor of tumour-infiltrating lymphocytes
WES: Whole exome sequencing
